# Rapid detection of porins by matrix-assisted laser desorption/ionization-time of flight mass spectrometry

**DOI:** 10.3389/fmicb.2015.00784

**Published:** 2015-08-04

**Authors:** Yan-Yan Hu, Jia-Chang Cai, Hong-Wei Zhou, Rong Zhang, Gong-Xiang Chen

**Affiliations:** Second Affiliated Hospital of Zhejiang University, Zhejiang University, HangzhouChina

**Keywords:** outer membrane protein, MALDI-TOF MS, SDS-PAGE, *E. coli*, *K. pneumoniae*

## Abstract

The rapid and cost-efficient determination of carbapenem resistance is an important prerequisite for the choice of an adequate antibiotic therapy. A MALDI-TOF MS-based assay was set up to detect porins in the current study. A loss of the components of porin alone such as OmpK35/OmpK36 or together with the production of carbapenemases will augment the carbapenem resistance. Ten strains of *Escherichia coli* and eight strains of *Klebsiella pneumoniae* were conducted for both sodium dodecylsulfate-polyacrylamide gel electrophoresis (SDS-PAGE) and MALDI-TOF MS analysis. MALDI-TOF/TOF MS analysis was then performed to verify the correspondence of proteins between SDS-PAGE and MALDI-TOF MS. The results indicated that the mass spectrum of ca. 35,000, 37,000, and 38,000-m/z peaks of *E. coli* ATCC 25922 corresponded to OmpA, OmpC, and OmpF with molecular weight of approximately ca. 38, 40, and 41 kDa in SDS-PAGE gel, respectively. The band of OmpC and OmpF porins were unable to be distinguished by SDS-PAGE, whereas it was easy to be differentiated by MALDI-TOF MS. As for *K. pneumoniae* isolates, the mass spectrum of ca. 36,000 and 38,600-m/z peaks was observed corresponding to OmpA and OmpK36 with molecular weight of approximately ca. 40 and 42 kDa in SDS-PAGE gel, respectively. Porin OmpK35 was not observed in the current SDS-PAGE, while a 37,000-m/z peak was found in *K. pneumoniae* ATCC 13883 and carbapenem-susceptible strains by MALDI-TOF MS which was presumed to be the characteristic peak of the OmpK35 porin. Compared with SDS-PAGE, MALDI-TOF MS is able to rapidly identify the porin-deficient strains within half an hour with better sensitivity, less cost, and is easier to operate and has less interference.

## Introduction

Carbapenems, which are effective against extended spectrum β-Lactamases (ESBLs) or AmpC-producing strains, are considered to be the most active antibiotics against ESBLs-producing Enterobacteriaceae and are thus extensively used for the treatment of serious infections especially the infections caused by multi-drug resistant Enterobacteriaceae (Baldwin et al., 2008). However, abusing of antibiotics is becoming an increasingly serious problem which followed by emergence of carbapenem resistance. Thus, a rapid detection and determination method of the drug resistance mechanism is urgently needed in response to the growing number of carbapenem-resistant Enterobacteriaceae. According to the reports (Queenan and Bush, 2007; Liu et al., 2008; Papp-Wallace et al., 2011; Nagaraj et al., 2012), the predominant carbapenem resistant mechanisms in Enterobacteriaceae are attributed to the following two aspects: (1) production of carbapenemases; (2) combination of high-level production of ESBLs or AmpC β-lactamases together with loss of porin. Porins allow carbapenems to diffuse into the bacterial, thus, missing or decreased expression of porins lead to carbapenem resistance against bacterial. The most relevant porins with carbapenem-resistance are OmpC and OmpF in *Escherichia coli* and OmpK35 and OmpK36 in *Klebsiella pneumoniae* (Nikaido et al., 1983; Alberti et al., 1995; Domenech-Sanchez et al., 2003).

Matrix-assisted laser desorption/ionization time of flight mass spectrometry (MALDI-TOF MS), a recently introduced technique for microorganism identification (Bizzini and Greub, 2010; Wieser et al., 2012) has also been applied for the rapid detection of antibiotic susceptibility. MALDI-TOF MS-based assay for the detection of β-lactamases especially carbapenemases activity had been described (Burckhardt and Zimmermann, 2011; Hrabak et al., 2011; Sparbier et al., 2012). β-lactamases producing strains can be rapidly detected through comparing the characteristic peaks of β-lactam antibiotics with peaks after incubation of antibiotics together with bacteria. The characteristic spectral peaks of antibiotics would disappear if the bacteria produce β-lactamases. The average turn-around time of the method is approximately 3~4 h, much faster than Modified Hodge Test (MHT) which is recommend by CLSI for carbapenem detection and takes for about 16~18 h (CLSI, 2014). Besides this, MALDI-TOF MS had been used in rapid detection of other drug-resistance such as methicillin resistant *Staphylococcus aureus* (Edwards-Jones et al., 2000), vancomycin-resistant *Enterococcus* spp. (Griffin et al., 2012) and rifampin or isoniazid resistant *Mycobacterium tuberculosis* (Ikryannikova et al., 2007). Though the MALDI-TOF MS has been successfully used in detection of antibiotic-resistance, few reports of rapid detection of porins was reported. Though LC-MS/MS (Prajanban et al., 2012) or even LC-MALDI MS (Liu et al., 2011) were already used for protein identification and characterization in analytical chemistry, these devices cost much higher than MALDI-TOF MS and were only limited for laboratory research not for microorganism identification. Porins are usually detected using the classical sodium dodecylsulfate-polyacrylamide gel electrophoresis (SDS-PAGE) method, and it is laborious, and time-consuming. Therefore, we have applied the method for the rapid detection of porins in *K. pneumoniae* (Cai et al., 2012).

In the current study, analysis of porins from 18 isolates including 10 strains of *E. coli* and 8 strains of *K. pneumoniae* were conducted by MALDI-TOF MS. MALDI-TOF/TOF MS was then performed to identify the bands in the SDS-PAGE gel and correlate them with the proteins observed in MS.

## Materials and Methods

### Bacterial Strains

A total of 18 non-duplicated Enterobacteriaceae strains including one carbapenem susceptible *E. coli* isolate, ATCC 25922, eight *E. coli* isolates with carbapenem resistance or reduced susceptibility, one carbapenem susceptible *K. pneumoniae* isolate, ATCC 13883, and six *K. pneumoniae* isolates with carbapenem resistance or reduced susceptibility were selected in this study (**Table 1**). Species identification for the 18 isolates were initially performed with the Vitek 2 compact system (bioMérieux, Durham, NC, USA) and then confirmed by MALDI-TOF MS (Bruker Daltonik GmbH, Bremen, Germany; MALDI Biotyper 3.0).

**Table 1:** Carbapenem susceptibility and carbapenemase and β-lactamase genes of the selected 18 strains.

Strain*	Previous name	β-lactamases	IPM^#^ (μg/ml)	MEM (μg/ml)	ETP (μg/ml)	Reference
EC1	—	CMY-2, CTX-M-14, TEM-1	16	16	32	This study
EC2	E21	KPC-2, TEM-1	16	16	32	Cai et al. (2014)
EC3	—	CTX-M-14	1	2	8	This study
EC4	E6	KPC-2, CTX-M-15	8	16	64	Cai et al. (2014)
EC5	E1	KPC-2, CTX-M-15	2	2	8	Cai et al. (2014)
EC6	—	TEM-1, CTX-M-55, CMY-2	2	1	8	This study
EC7	—	TEM-1, CTX-M-15, CMY-2	16	32	128	This study
EC8	—	TEM-1, CTX-M-14, DHA-1, SHV-12	8	8	16	This study
EC9	—	—	≤0.125	≤0.125	≤0.125	This study
KP1	Z2110	TEM-1, SHV-11, CTX-M-14, DHA-1	4	2	32	Cai et al. (2012)
KP2	Z2554	TEM-1, SHV-11, CTX-M-14	0.5	8	16	Cai et al. (2012)
KP3	Z4	IMP-4, TEM-1, SHV-1	32	32	256	Cai et al. (2012)
KP4	Z5	IMP-4, TEM-1, SHV-12	1	1	2	Cai et al. (2012)
KP5	K1	KPC-2, TEM-1, SHV-11, CTX-M-14	4	4	8	Cai et al. (2012)
KP6	K10	KPC-2, TEM-1, SHV-12, CTX-M-14	128	256	>256	Cai et al. (2012)
KP7	S1	—	≤0.125	≤0.125	≤0.125	Cai et al. (2012)
ATCC 25922	—	—	≤0.125	≤0.125	≤0.125	This study
ATCC 13883	—	—	≤0.125	≤0.125	≤0.125	This study

*EC1–EC8, clinically isolated *E. coli* strains with carbapenem resistance or reduced susceptibility; EC9, clinically isolated *E. coli* strain with carbapenem-susceptible; KP1–KP6, clinically isolated *K. pneumoniae* strains with carbapenem-resistance or reduced susceptibility; KP7, clinically isolated *K. pneumoniae* strain with carbapenem-susceptible.

*^#^IPM, imipenem; MEM, meropenem; ETP, ertapenem.*

### Antimicrobial Susceptibility Testing

The minimum inhibitory concentrations (MICs) of imipenem, meropenem, and ertapenem were determined by Mueller-Hinton (M-H) agar dilution method and were interpreted in accordance with the standards of Clinical Laboratory Standards Institute (CLSI; CLSI, 2013), *E. coli* ATCC 25922 and *K. pneumoniae* ATCC 13883 were used for quality control.

### PCR Amplification

Screening for common ESBLs or carbapenemase genes, including *bla*_TEM_ (Yu et al., 2007), *bla*_SHV_ (Yu et al., 2007), *bla*_CTX-M_ (Yu et al., 2007), AmpC (Perez-Perez and Hanson, 2002), *bla*_KPC_ (Yigit et al., 2001), *bla*_IMP_ (Queenan and Bush, 2007), *bla*_V IM_ (Queenan and Bush, 2007), and *bla*_NDM_ (Zhang et al., 2013) were performed by PCR amplification using specific primers in a Tpersonal Cycler (Biometra, Germany). The PCR products were then sequenced using an ABI3730 Sequencer (Applied Biosystems, Foster City, CA, USA), and compared with the reported sequences from GenBank.

### Analysis of Outer Membrane Proteins (OMPs)

Outer membrane proteins (OMPs) were extracted as described previously (Hernandez-Alles et al., 1999). OMPs were solubilized in electrophoresis sample buffer and boiled for 5 min before electrophoretic analysis. Strains were grown overnight in nutrient broth without NaCl at 37°C with shaking. OMPs were determined by SDS-PAGE with a 5% stacking gel (4.86% acrylamide/0.17% bisacrylamide/0.1% SDS) and a 15% separating gel (14.5% acrylamide/0.5% bisacrylamide/0.1% SDS) containing 20% urea. The 0.75 mm thickness gel was run at a constant current of 20 mA for 140 min with a Mini Protein 3 slab electrophoresis cell (Bio-Rad). OMPs were detected with Coomassie brilliant blue R-250 staining.

### MALDI-TOF MS Analysis

One microliter of the extracted OMPs was applied onto a polished steel 96-spot target (Bruker Daltonik GmbH, catalog: 224989) and after drying at room temperature, 1 μl of 20 mg/ml 2,5-dihydroxybenzoic acid matrix (DHB, dissolved in 50% ACN+2.5% TFA) was added to each target spot. MALDI-TOF MS was performed with the flexControl 3.3 software (Bruker Daltonik GmbH) operating in positive linear ion mode between 5,000 and 50,000 Da. The protocol used in this study had a slight modification on LP_66KDa.par (provided by the manufacturer). The main parameters were set as follows: mode, low range; ion source 1, 19.00 kV; ion source 2, 17.20 kV; lens, 6.00 kV; pulsed ion extraction, 0 ns; digitizer trigger level, 1000 mV; laser range 30%, laser offset 15–30%, laser frequency, 60 Hz; linear 13.0X, Sample rate 2.0; electronic gain: enhanced 100 mV; shots: 100, and 500 shots were summed up for each sample. The protein calibration standard II (Bruker Daltonik GmbH) with the significant peaks of ca. 22,307.0, 23,982.0, 33,216.0, 44,613.0, 66,431.0, 89,225.0, and 132,861.0 m/z Dalton was used for calibration, and the calibration error was regulated under 300 ppm strictly. The mass spectra were analyzed using Bruker daltonics flexAnalysis 3.3 software.

### Tryptic in-Gel Digestion and MALDI-TOF/TOF MS Analysis

The OMP bands were carefully excised and transferred into a 1.5 ml Eppendorf centrifuge tube. Gel chips were destained with 30 μM NH_4_HCO_3_/30% ACN. After freeze-drying, the gels were hydrated in 5 μL (2.5 ng/μL) trypsin solution at 4°C for 60 min and then 30 μL of 25 mM NH_4_HCO_3_ buffer was added and incubated at 37°C overnight. The supernatant was directly applied onto the sample plate for MALDI-TOF MS analysis. MALDI-TOF/TOF MS analysis was performed using a Bruker Autoflex Speed MALDI-TOF/TOF MS (Bruker Daltonics, Bremen, Germany) operated in the reflector mode for MALDI-TOF peptide mass fingerprint (PMF) followed by LIFT mode for tandem MS in a fully automated approach using the FlexControl^TM^ software. Samples were analyzed by one PMF from MALDI-TOF, followed by additional LIFT-TOF-TOF MS/MS analysis of the top 7–10 highest intensity peptides. Data were accumulated from 1000 consecutive laser shots to produce PMF followed by 200 precursor ion scans and 2000 MS/MS spectra in LIFT mode. Calibration for PMF samples (digests) was performed both externally on an adjacent spot for each sample using a mixture of nine peptides ranging from m/z 757.40 to 3147.47. The m/z range was 700–4000 for PMF and dynamically adjusted for each ion in MS/MS. PMF and MS/MS spectra were interpreted primarily with the FlexAnalysis^TM^ software (Bruker Daltonics). Signal-to-noise ratio threshold was set to three. Blank gel was used as blank control through the entire steps.

### Database Searching of MALDI-TOF/TOF MS

For protein identification, database searches were carried out with MASCOT version 2.3 (Matrix Science, London, UK) against NCBInr database and were performed via BioTools 3.2 software (Bruker Daltonics). Species were restricted to bacteria (Eubacteria). The parameters used are: a specified trypsin enzymatic cleavage with one possible missed cleavage, monoisotopic masses, a mass tolerance of ±50 ppm for the parent ion and ±0.7 Da for the fragment ion. Carbamidomethylation of cysteine residues as fixed modification, oxidation of methionine as differential modifications. The protein identification was inferred from peptide-spectrum matches by assembling the identified peptide sequences into proteins.

## Results

### Antimicrobial Susceptibility and DNA Sequence Analysis

Minimum inhibitory concentrations and β-lactamase genes of the 18 strains were listed in **Table 1**. In addition to two susceptible clinical isolates and two ATCC strains, the other 14 isolates showed reduced susceptibility or resistance to carbapenems. β-lactamase genes were detected in all the 14 strains, five of them produced KPC-2-type carbapenemase, and two of them produced IMP-4-type carbapenemase (**Table 1**).

### Sodium Dodecylsulfate-Polyacrylamide Gel Electrophoresis

**FIGURE 1:**
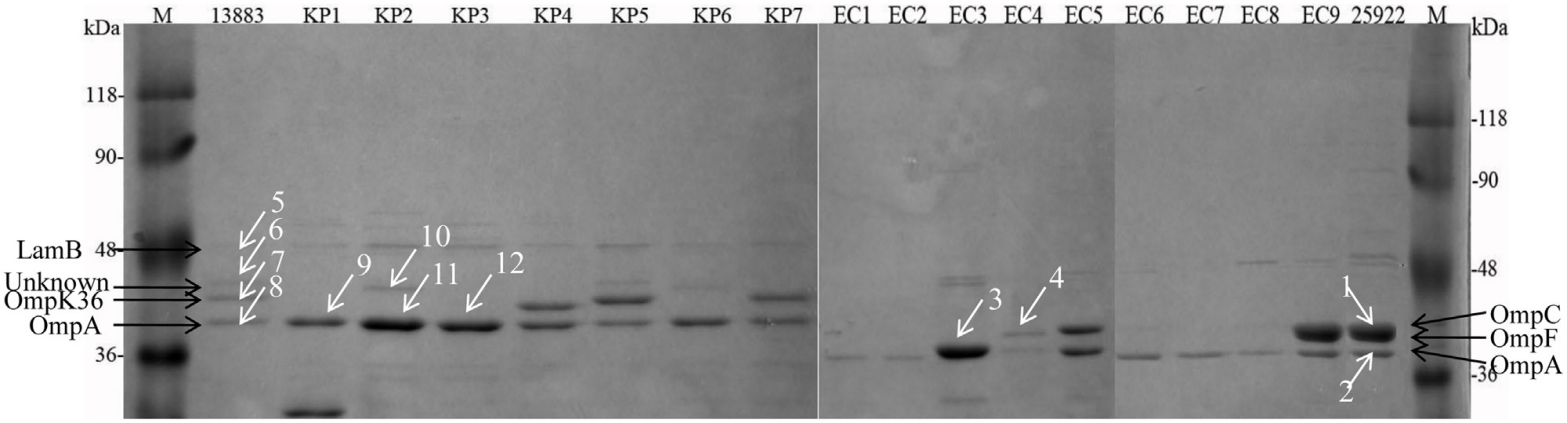
**Sodium dodecylsulfate-polyacrylamide gel electrophoresis (SDS-PAGE) analysis of OMPs extracted from 8 *K. pneumoniae* and 10 *E. coli* isolates.** M, protein molecular weight marker; KP1–KP6, carbapenem-resistant or reduced susceptible *K. pneumoniae* isolates; KP7, carbapenem-susceptible clinically isolated *K. pneumoniae*; EC1–EC8, carbapenem-resistant or reduced susceptible *E. coli* isolates; EC9, carbapenem-susceptible clinically isolated *E. coli*; 13883 and 25922 represent for ATCC strains of *K. pneumoniae* and *E. coli*, respectively. Twelve white arrowed bands were digested with trypsin for the following MALDI-TOF/TOF MS analysis.

The arrows observed in the ATCC strains between molecular weight of 36–48 kDa were assigned to 48 kDa-LamB, 44 kDa-putative OMP, 42 kDa-OmpK36, and 40 kDa-OmpA for *K. pneumoniae* isolates and 41 kDa-OmpC, 40 kDa-OmpF, and 38 kDa-OmpA for *E. coli* isolates, respectively (**Figure 1**). Forty-one kDa-OmpC and 40 kDa-OmpF bands in *E. coli* ATCC 25922 were in close proximity and were difficult to be distinguished (**Figure 1**). OmpK35 was not found in the current study. *K. pneumoniae* KP1–KP3 and KP6 failed to express OmpK36. *E. coli* EC1–EC3, EC6–EC8 failed to express OmpC and OmpF; *E. coli* EC4 and EC5 failed to express OmpF.

### MALDI-TOF MS Analysis

**FIGURE 2:**
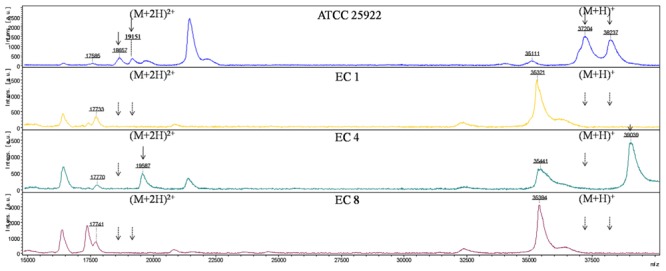
**MALDI-TOF MS analysis of *E. coli* isolates**. x axis, mass per charge in Daltons (m/z, Da); y axis, absolute intensity of signal. The solid arrows represent peaks with approximately 37,000- and 38,000-m/z corresponding to OmpC and OmpF, and their corresponding peaks of 18,500- and 19,000-m/z with (M+2H)^2+^. The dotted arrows indicate the loss of 18,500-, 19,000-, 37,000- and 38,000-m/z peaks. 35,000-m/z peak was detected in all the strains representing for OmpA.

Six major peaks with m/z of ca. 17,500, 18,500, 19,000, 35,000, 37,000, and 38,000, respectively, were detected in *E. coli* ATCC 25922 which expressed porins as analyzed by SDS-PAGE. The ca. -17,500, 18,500, and 19,000-m/z peaks (M+2H)^2+^ observed in some strains were considered to be the multiple charged state of the ca. -35,000, 37,000, and 38,000-m/z peaks. The ca. -35,000, 37,000, and 38,000-m/z peaks were presumed to be OmpA, OmpF, and OmpC with ~38, 40, and 41 kDa in SDS-PAGE gel, respectively. For the *E. coli* EC1–EC3, EC6–EC8 isolates which failed to express OmpC and OmpF porins, the peaks of ca. -37,000 and 38,000-m/z disappeared along with the (M+2H)^2+^ peaks ca. -18,500 and 19,000-m/z. Similarly, the ca. -37,000-m/z disappeared along with the (M+2H)^2+^ peak ca. -18,500-m/z mass spectra in the OmpF-deficient *E. coli* EC4 and EC5 isolates (**Figure 2**).

**FIGURE 3:**
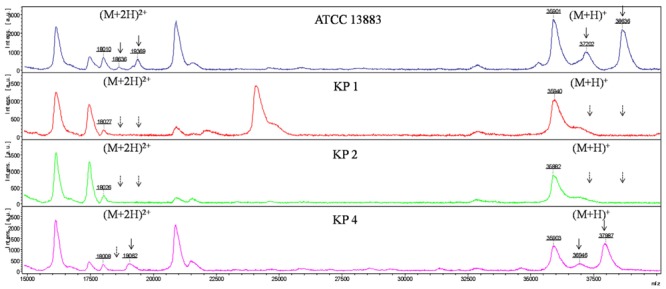
**MALDI-TOF MS analysis of *K. pneumoniae* isolates**. x axis, mass per charge in Daltons (m/z, Da); y axis, absolute intensity of signal. The solid arrows represent peaks with approximately 37,000- and 38,600-m/z corresponding to OmpK35 and OmpK36, and their corresponding peaks of 18,500- and 19,300-m/z with (M+2H)^2+^. The dotted arrows indicate the loss of 18,500-, 19,300-, 37,000-, and 38,600-m/z peaks. 36,000-m/z peak was detected in all the strains representing for OmpA.

As with *E. coli* isolates, six major peaks with m/z of ca. 18,000, 18,500, 19,000, 36,000, 37,000, and 38,600, respectively, were observed in *K. pneumoniae* ATCC 13883, KP4, KP5, and KP7. The ca. -18,000, 18,500, and 19,300-m/z peaks (M+2H)^2+^ observed in some strains were considered to be the multiple charged state of the ca. -36,000, 37,000, and 38,600-m/z peaks. The ca. -36,000 and 38,600-m/z peaks were presumed to be OmpA and OmpK36 with ~40 and 42 kDa in SDS-PAGE, respectively. The ca. -37,000-m/z peak was presumed to be OmpK35 which was not detected by SDS-PAGE. The intensity of signal of the ca. -37,000-m/z peak was much lower than the ca. -36,000 and 38,600-m/z peaks. For the OmpK36-deficient isolates, the peak of ca. -38,600-m/z disappeared along with the (M+2H)^2+^ peak ca. -19,300-m/z. Mass spectrum of *K. pneumoniae* ATCC 13883 (similar with KP5 and KP7), KP1 (similar with KP3 and KP6), KP2 and KP4 were displayed in **Figure 3**. Forty-eight kDa-LamB and 44 kDa-putative OMP observed in SDS-PAGE of some strains were not detected in MALDI-TOF MS, maybe due to the large molecular weight or low intensity of signal.

### MALDI-TOF/TOF MS Analysis

Twelve protein bands were successfully identified by MALDI-TOF/TOF MS after in-gel tryptic digestion. These protein bands represented for well-known proteins such as OmpA, OmpC, OmpF, OmpK36, and LamB. Bands 2, 3, 8, 9, 11, 12 were identified by peptide mass sequence analysis as OmpA; Band 1 was identified as a mixture of OmpC and OmpF, and Band 4 was identified as OmpC. Band 7 matched well to OmpK36 (score 441, sequence coverage 24%). However, bands 6 and 10 which might be mistaken for OmpK35 were identified as putative OMP. Mass spectrum of tryptic-digested and MALDI-TOF/TOF MS analyzed OmpA, OmpC, OmpK36 and putative OMP were showed in Supplementary Figures S1–S8. None of the OmpK35 bands were found in the current study. The names of the proteins, MASCOT scores, peptide sequences and the specific correspondence between SDS-PAGE, MALDI-TOF MS and MALDI-TOF/TOF MS were indicated in **Table 2**.

**Table 2:** Proteins identified by MALDI-TOF/TOF MS after SDS-PAGE.

Strain	Spot no.	SDS-PAGE band	Mass spectra (m/z)	Match to	Protein name	Score	Nominal mass	Calculated pI	Sequence coverage	Peptide sequences
ATCC25922	1	40 and 41 kDa*	37,000 and 38,000	gi|15832358	OmpC	171	40483	4.55	8%	R.GNGFATYR.N K.FQDVGSFDYGR.N K.INLLDDNQFTR.D
				gi|486377872	OmpF	62	39174	4.76	3%	K.YADVGSFDYGR.N
ATCC25922	2	38 kDa	35,000	gi|215486075	OmpA	450	37176	5.99	16%	K.GIPADKISAR.G K.DNTWYTGAK.L K.DGSVVVLGYTDR.I R.RAQSVVDYLISK.G R.IGSDAYNQALSER.R R.IGSDAYNQALSERR.A
EC3	3	38 kDa	35,000	gi|15800816	OmpA	583	37178	5.99	20%	K.GIPADKISAR.G K.LGYPITDDLDIYTR.L K.DGSVVVLGYTDR.I R.IGSDAYNQGLSER.R R.IGSDAYNQGLSERR.A R.RAQSVVDYLISK.G K.GIPADKISAR.G
EC4	4	40 or 41 kDa^#^	38,000	gi|241110696	OmpC	217	41394	4.55	8%	K.FQDVGSFDYGR.N R.TDDQNFGLNGYGER.Y R.VAFAGLKFQDVGSFDYGR.N
ATCC13883	5	48 kDa	ND^||^	gi|490199258	LamB	217	47725	4.83	14%	K.EGDKSFYFDTNVAYSVSQQNDWESTSPAFR.E R.VYDNIVPNDVFDVR.L K.VTLAQQWQAGDSIWSRPAIR.V
ATCC13883	6	44 kDa	ND	gi|152969047	putative omp	118	43185	5.29	6%	K.QDPQAGDPLSR.L K.FPINQYAVK.S R.LRYDF.-
ATCC13883	7	42 kDa	38,600	gi|221192800	OmpK36	441	40816	4.57	24%	K.FGDAGSFDYGR.N K.INLLDENDFTR.R K.YVDVGATYYFNK.N K.INLLDENDFTRR.A R.FSGNGESDSISGFANK.A K.YDANNIYLATQYTQTYNATR.F K.IDGLHYFSDDKSVDGDQTYMR.V
ATCC13883	8	40 kDa	36,000	gi|3915781	OmpA	429	37152	5.73	25%	K.LGYPITDDLDIYTR.L R.ADSKGNYASTGVSR.S R.FGQEDAAPVVAPAPAPAPEVATK.H K.DGSAVVLGYTDR.I R.IGSEAYNQQLSEKR.A K.RAQSVVDYLVAK.G
KP1	9	40 kDa	36,000	gi|152969543	OmpA	265	38021	6.00	16%	K.LGWSQYHDTGFYGNGFQNNNGPTR.N K.LGYPITDDLDIYTR.L R.SEHDTGVSPVFAGGVEWAVTR.D
KP2	10	44 kDa	ND	gi|152969047	putative omp	118	43185	5.29	6%	K.QDPQAGDPLSR.L K.FPINQYAVK.S R.LRYDF.-
KP2	11	42 kDa	36,000	gi|152969543	OmpA	265	38021	6.00	16%	K.LGWSQYHDTGFYGNGFQNNNGPTR.N K.LGYPITDDLDIYTR.L R.SEHDTGVSPVFAGGVEWAVTR.D
KP3	12	42 kDa	36,000	gi|152969543	OmpA	265	38021	6.00	16%	K.LGWSQYHDTGFYGNGFQNNNGPTR.N K.LGYPITDDLDIYTR.L R.SEHDTGVSPVFAGGVEWAVTR.D

*Forty-one kDa-OmpC and 40 kDa-OmpF bands of *E. coli* ATCC25922 in SDS-PAGE were in close proximity and were difficult to cut apart.

*^#^It is difficult to identify *E. coli* EC4 as 40 kDa-OmpF deficiency or 41 kDa-OmpC deficiency through SDS-PAGE.*

*^||^ND, not detected.*

## Discussion

MALDI-TOF MS is an emerging method where material is ionized in a high vacuum chamber and accelerated in an electric field. The fragment size (m/z) can be inferred from the time of flight of the ionized fragments. In the last 2 years MALDI-TOF MS had became a rapid detection tool for carbapenemases and other β-lactamases, which has significantly reduced the detection time to about 4 h (Burckhardt and Zimmermann, 2011; Sparbier et al., 2012). We previously reported the rapid detection of porins by MALDI-TOF MS in *K. pneumoniae* isolates (Cai et al., 2012). Our current study extended the species to *E. coli* isolates and further confirmed the specific correspondences between SDS-PAGE and MALDI-TOF MS.

Combining with SDS-PAGE, MALDI-TOF MS and MALDI-TOF/TOF MS, we obviously observed that the mass spectrum of ca. 35,000, 37,000, and 38,000-m/z peaks of *E. coli* ATCC 25922 corresponded to OmpA, OmpC, and OmpF with molecular weight of ca. 38, 40, and 41 kDa in SDS-PAGE, respectively. OmpC and OmpF are reported to be related to carbapenem-resistance and OmpF migrates slightly faster than OmpC (Nikaido et al., 1983). They were difficult to be distinguished in the SDS-PAGE gel after a 2.5 h electrophoresis in the current study. Nevertheless, they could be clearly distinguished by MALDI-TOF MS through the m/z of ca. 37,000 and 38,000. Meanwhile, it was hard to determine the *E. coli* EC4 and EC5 strains as OmpC-deficient or OmpF-deficient isolates through SDS-PAGE. However, via the mass spectra of *E. coli* EC4 and EC5, it was unambiguous that the ca. 37,000-m/z peak corresponded to OmpF. Accordingly, we can draw the conclusion that *E. coli* EC4 and EC5 were OmpF-deficient isolates. It follows that the resolution of MALDI-TOF MS is much higher than SDS-PAGE.

Porin OmpK36, whose amino acid sequence is very closer to that of *E. coli* OmpC porin and porin OmpK35 whose sequence is homologous to that of *E. coli* OmpF porin are the resistance-related porins in *K. pneumoniae* isolates (Alberti et al., 1995; Hernandezalles et al., 1995; Hernandez-Alles et al., 1999). The mass spectrum of ca. 36,000 and 38,600-m/z peaks of *K. pneumoniae* ATCC 13883 corresponded to OmpA and OmpK36 with molecular weight of ca. 40 and 42 kDa in SDS-PAGE, respectively. Porin OmpK35 was not observed in the current SDS-PAGE, while a 37,000-m/z peak was found in *K. pneumoniae* ATCC 13883 between 36,000 and 38,600-m/z peaks which might be the characteristic peak of OmpK35 porin. Observation of 37,000-m/z peak mass spectrum in *K. pneumoniae* ATCC 13883, KP4, KP5, and KP7 isolates, which was not found in the corresponding bands in the SDS-PAGE probably predicted that OmpK35 expressed at low level and was unable to be detected or it was difficult to be separated from OmpK36 within 2.5 h. The ESBLs-producing *K. pneumoniae* KP1, KP2, KP3, and KP6 were deficient in OmpK36 porin. Simultaneously, OmpK35 porin was not detected and the 37,000-m/z peak disappeared together with the 38,600-m/z peak which indicated that these isolates were deficient in both OmpK35 and OmpK36 porins. OmpK35 was not detected in all the isolates by SDS-PAGE, which result is in accordance with previous research which described that OmpK35 do not express or express at very low levels in the ESBLs-producing *K. pneumoniae* isolates and OmpK35 of 83% (24/29) isolates are undetectable by SDS-PAGE (Hernandez-Alles et al., 1999). While it is still inexplicable why *K. pneumoniae* ATCC 13833 lacks OmpK35 band, further study was needed.

In addition, *K. pneumoniae* ATCC 13883 and KP2 expressed a 44 kDa protein designated as a putative OMP. OmpK35 and OmpK36 migrates differently in various conditions. Hernandez-Alles et al. (1999) described that OmpK36 migrate faster than OmpK35. Therefore, the 44 kDa-putative OMP band might be easily mistaken for porin OmpK35 in the SDS-PAGE gel, whereas the high resolution of MALDI-TOF MS make it easy to observe the 37,000-m/z peak which was presumed to be the characteristic peak of OmpK35. Therefore, we can determine whether porin OmpK35 is expressed or not by MALDI-TOF MS rather than by SDS-PAGE.

The molecular weight of *K. pneumoniae* isolates of OmpK36 (42 vs. 38 kDa and OmpA (40 vs. 32 kDa) in the present study were slightly different with our previous reports (Cai et al., 2008; Wang et al., 2009). This phenomenon might probably be due to the difference of apparent molecular weight in the SDS-PAGE. Apparent molecular weight is normally different from the amino acids-based theoretical molecular weight. The apparent molecular weight is easily affected by many factors such as post-translational modification of proteins, electrophoresis conditions (PH, salt concentration) and the properties of proteins. All these factors might result in the discrepancy of apparent molecular weight with the theoretical molecular weight.

Moreover, Cornaglia et al. (1992) indicated that loss in either OmpC or OmpF did influence the susceptibility, although both were less important than the combined OmpC and OmpF deficiency. Meanwhile, Oteo et al. (2008) found that loss of OmpF did not affect the carbapenem susceptibility. The OmpF-deficient *E. coli* EC4 and EC5 isolates in present study produces KPC-type carbapenemase and CTX-M-type ESBLs, thus it is difficult to distinguish which factor(s) have resulted in the carbapenem resistance (production of carbapenemases or combination of high-level production of ESBLs together with porin loss or both). Whether porin OmpF played an important role in the carbapenem susceptibility requires further investigations.

The signal intensity of the ca. -37,000-m/z peak in carbapenem-susceptible strains was much lower than the ca. -36,000 and 38,600-m/z peaks. In accordance with the mass spectrum, the high intensity ca. -36,000 and 38,600-m/z peaks corresponding to OmpA and OmpK36 were able to be observed in SDS-PAGE gel, while the low intensity ca. -37,000-m/z peak corresponding to OmpK35 was undetectable by SDS-PAGE. Hence, we can presume that the intensity of signal might indirectly reflect the protein expression, which might expand the application of MALDI-TOF MS to assess the relative expression level of OMPs. Nevertheless, further study was needed to prove this point.

In summary, compared to SDS-PAGE, MALDI-TOF MS was able to rapidly identify the porin-deficient strains within half an hour. This method possesses better sensitivity, low cost, and is easier to operate and had less interference, therefore, it is suitable for application in general clinical laboratories.

## Conflict of Interest Statement

The authors declare that the research was conducted in the absence of any commercial or financial relationships that could be construed as a potential conflict of interest.
