# Supplementary material for: Rapid detection of porins by matrix-assisted laser desorption/ionization-time of flight mass spectrometry
Source: Front Microbiol. 2015 Aug 4;6:784. doi: 10.3389/fmicb.2015.00784 (PMC4524100; doi:10.3389/fmicb.2015.00784)
Supplement: Supplementary file 1 [file Image_1.PDF]

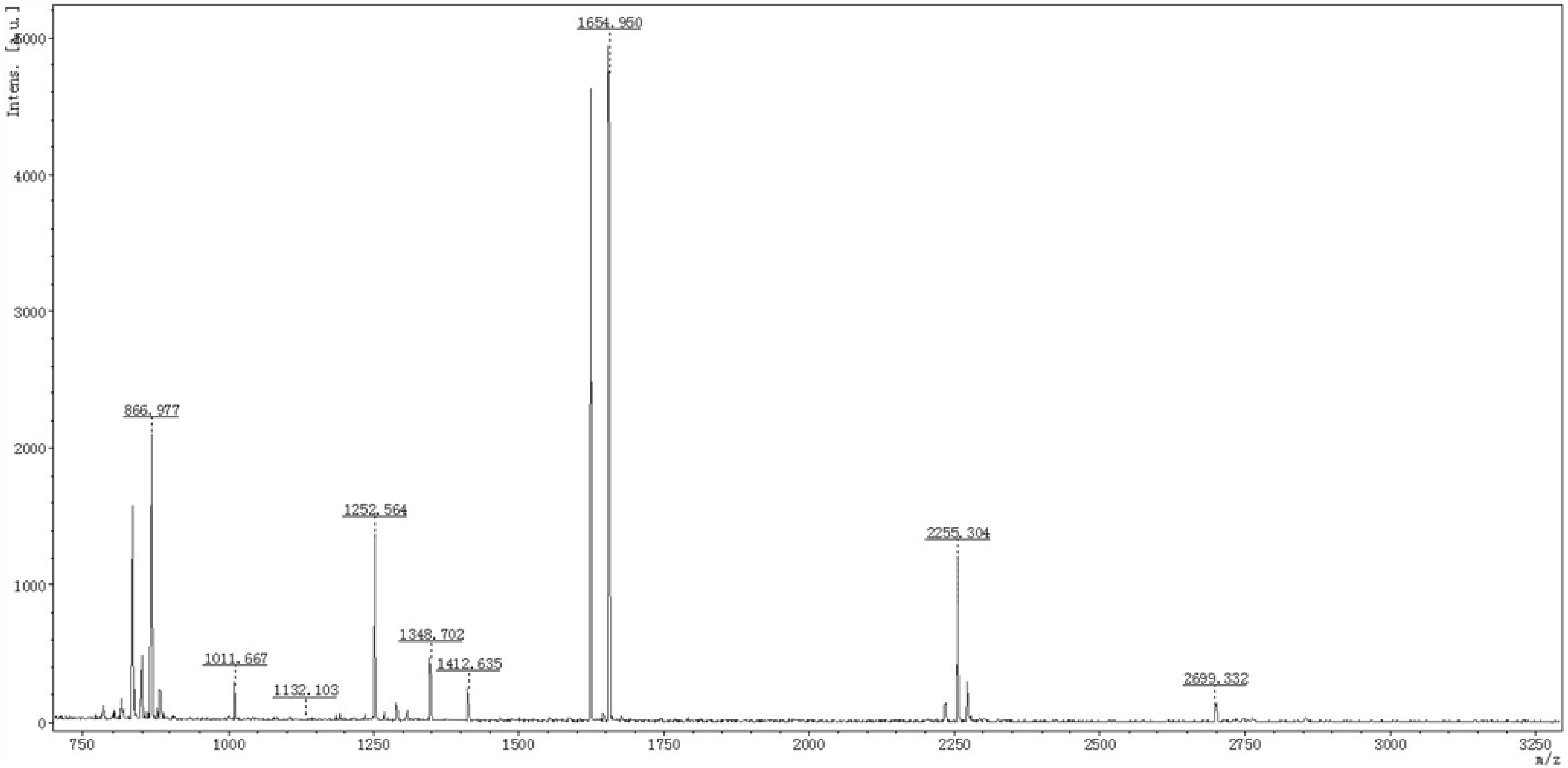


**Fig. S1** Mass spectrum of a trypsin-digested protein of OmpA isolated from *K. pneumoniae* ATCC 13883. 1,252.6, 1,348.7, 1,412.6, 1,622.9, 1,654.9, 2,233.3, 2,255.3 and 2,271.3-m/z peaks were chosed for the following MALDI-TOF/TOF MS analysis.

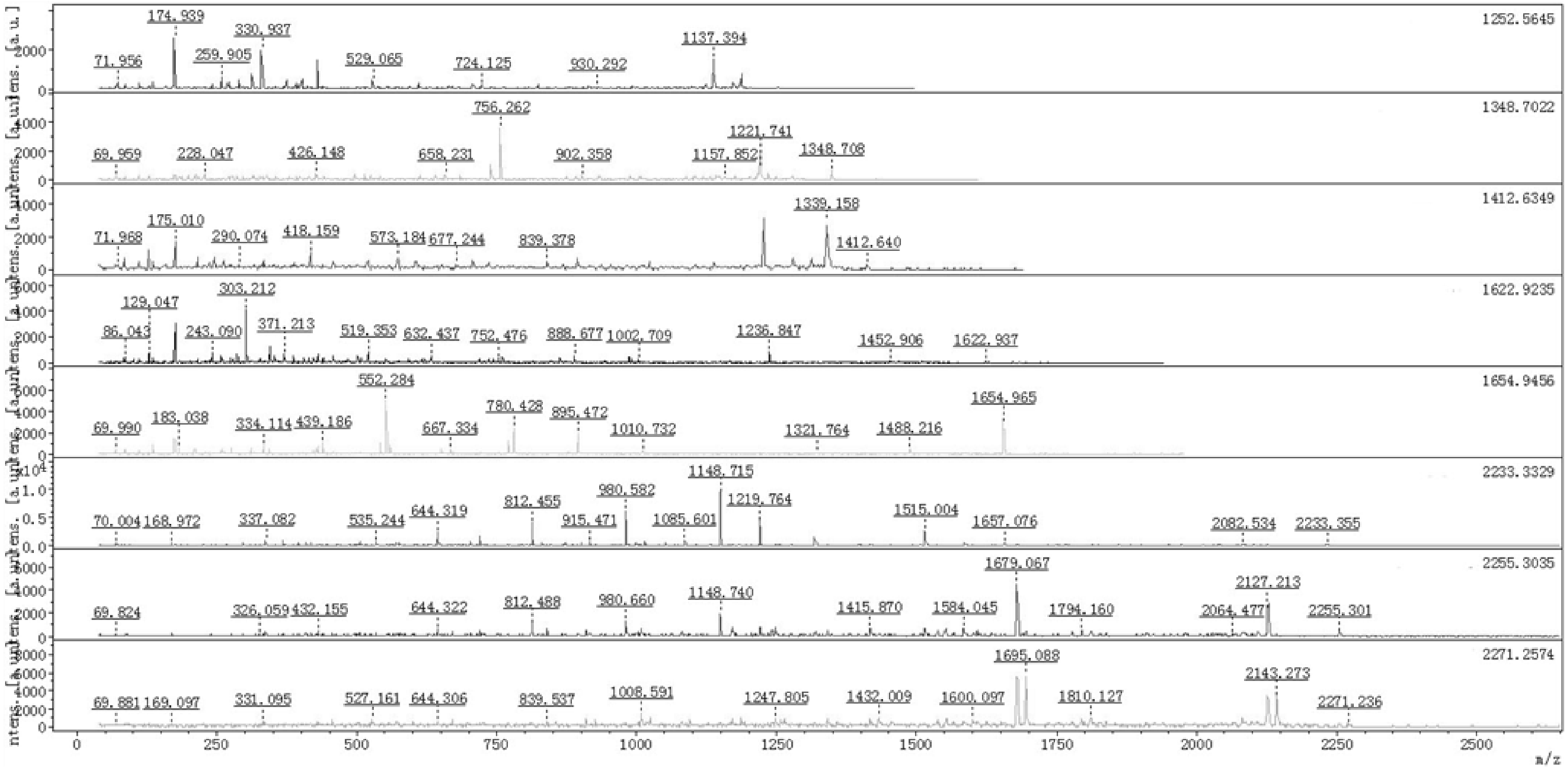


**Fig. S2** Mass spectra of eight tryptic peptides corresponding to OmpA of *K. pneumoniae* ATCC 13883 by MALDI-TOF/TOF MS.

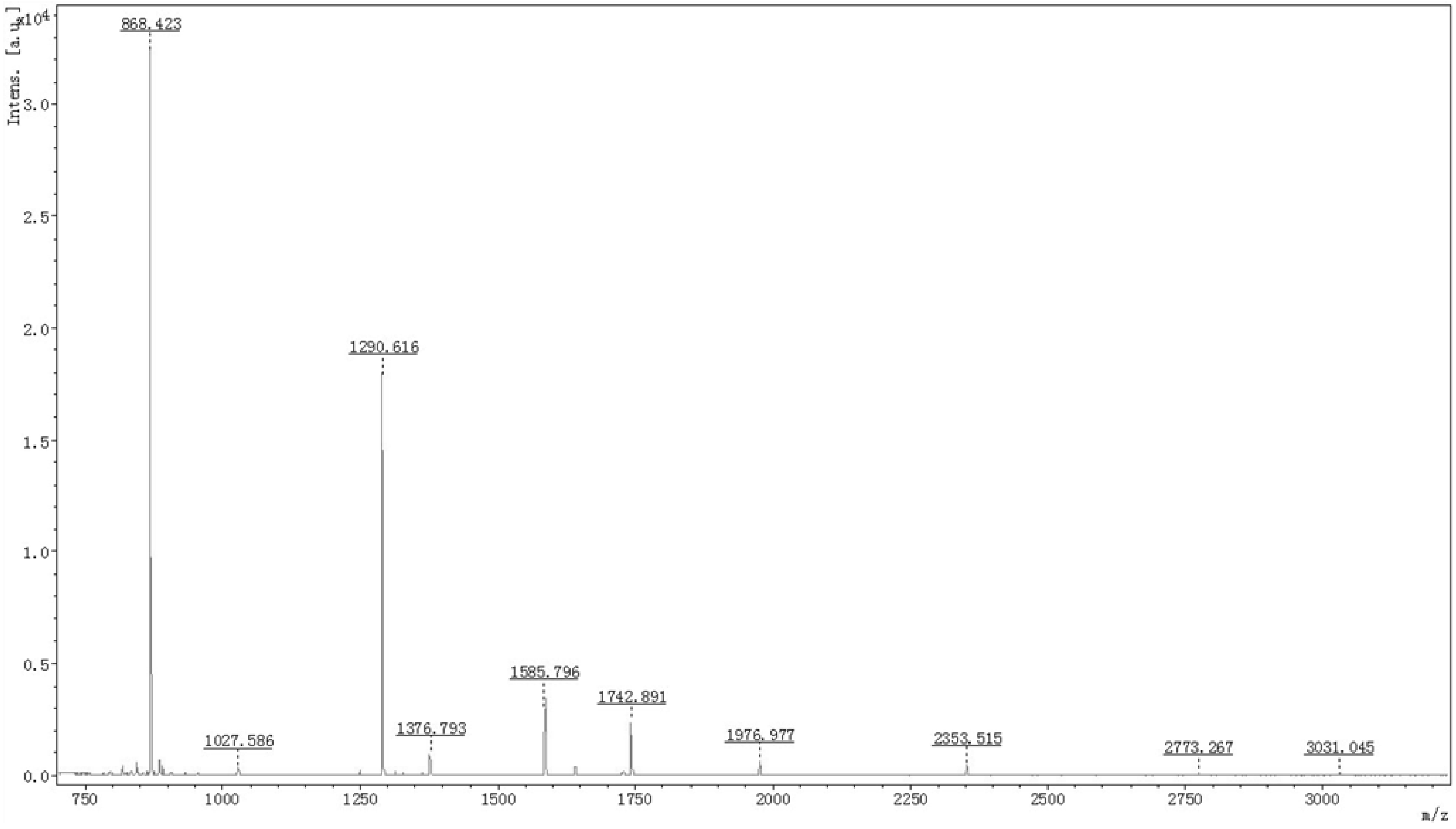


**Fig. S3** Mass spectrum of a trypsin-digested protein of OmpC isolated from *E.coli* EC4. 868.4, 1,290.6, 1,585.7, 1,640.8, 1,742.8, 1,976.7 and 2,352.6-m/z peaks were chosed for the following MALDI-TOF/TOF MS analysis.

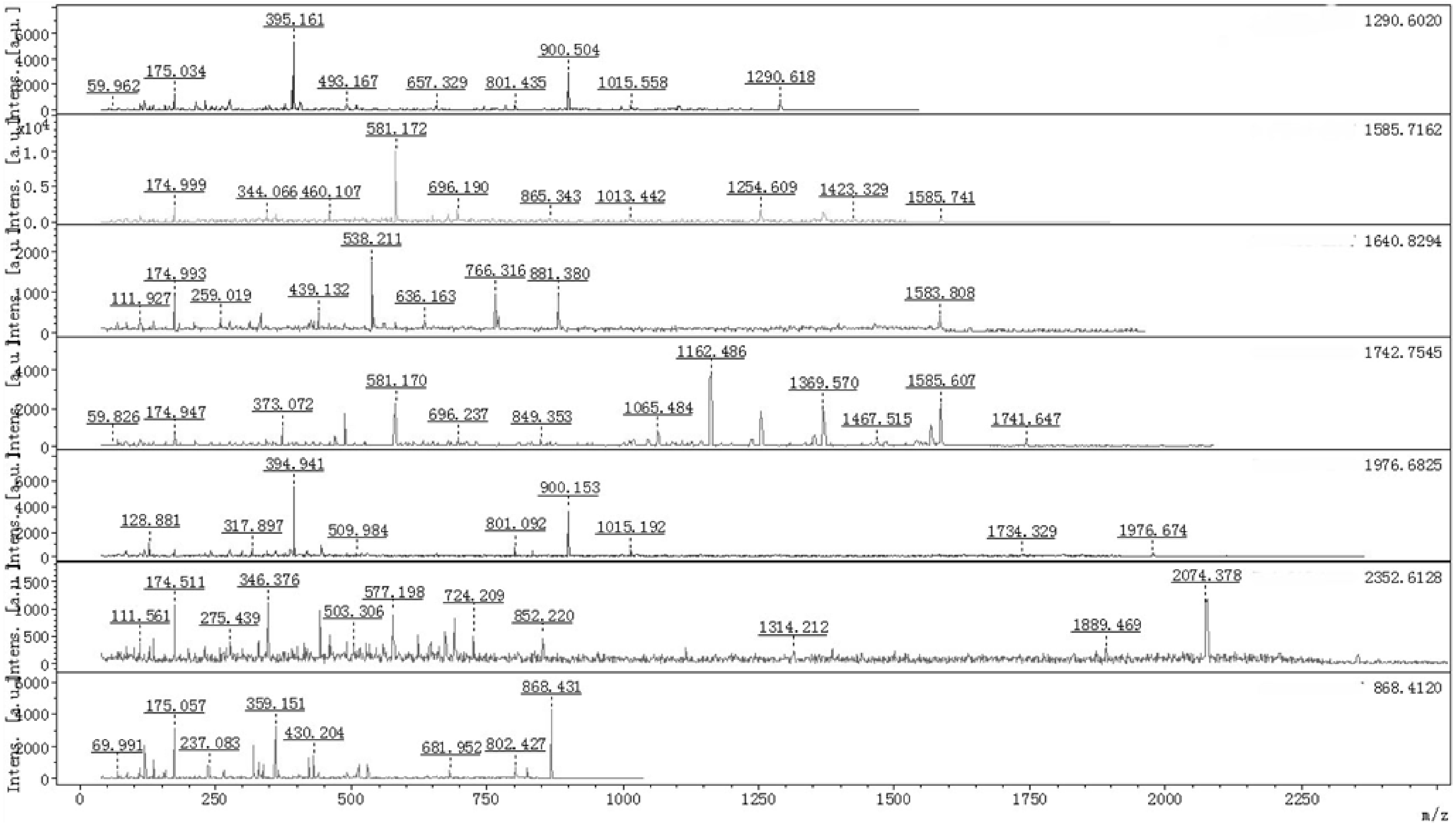


**Fig. S4** Mass spectra of seven tryptic peptides corresponding to OmpC of *E.coli* EC4 by MALDI-TOF/TOF MS.

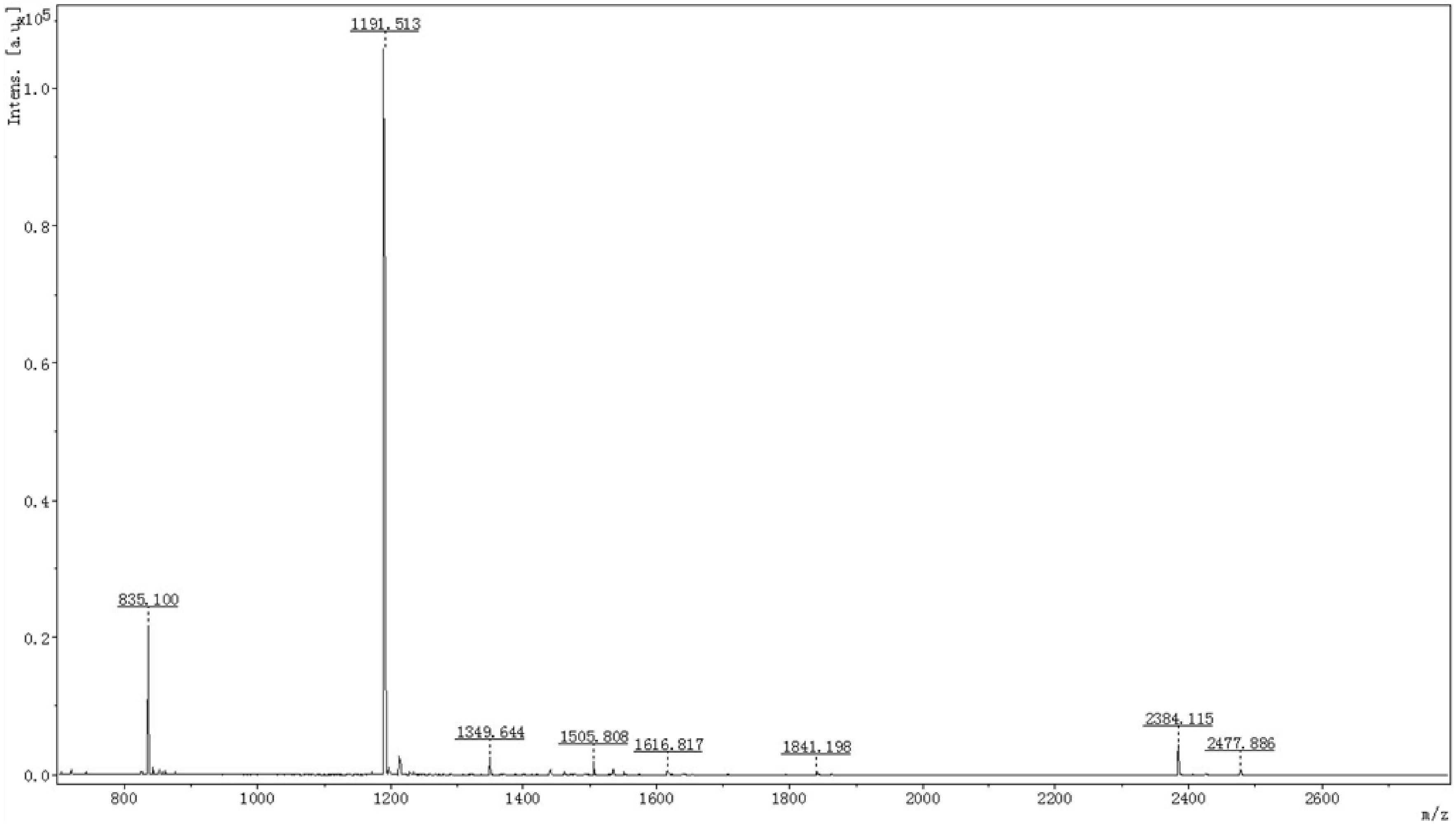


**Fig. S5** Mass spectrum of a trypsin-digested protein of OmpK36 isolated from *K. pneumoniae* ATCC 13883. 834.5, 851.5, 1,191.5, 1,349.7, 1,439.8, 1,505.9, 1,616.8, 1,841.0, 2,383.5 and 2,477.2-m/z peaks were chosed for the following MALDI-TOF/TOF MS analysis.

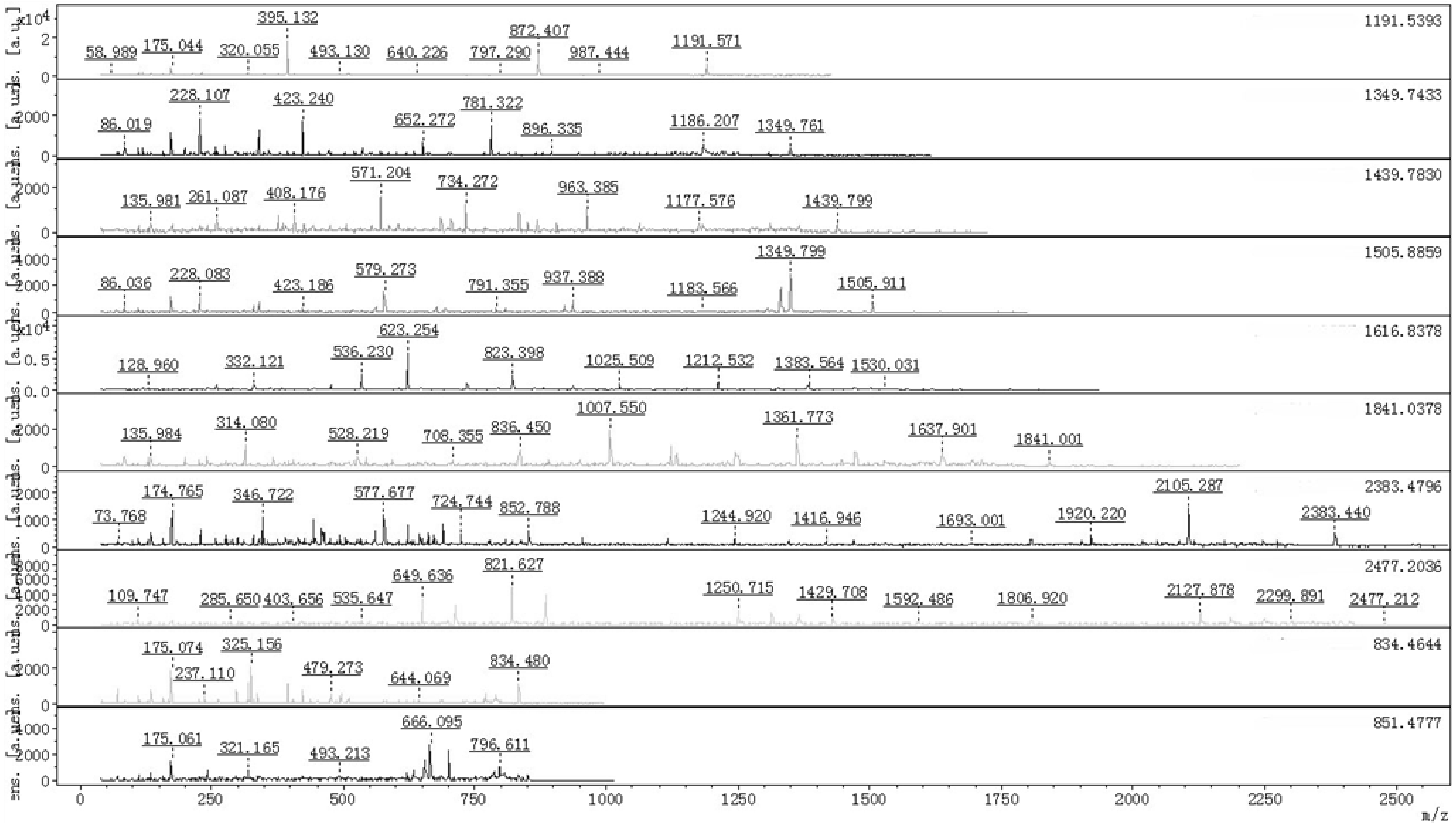

**Fig. S6** Mass spectra of ten tryptic peptides corresponding to OmpK36 of *K. pneumoniae* ATCC 13883 by MALDI-TOF/TOF MS.

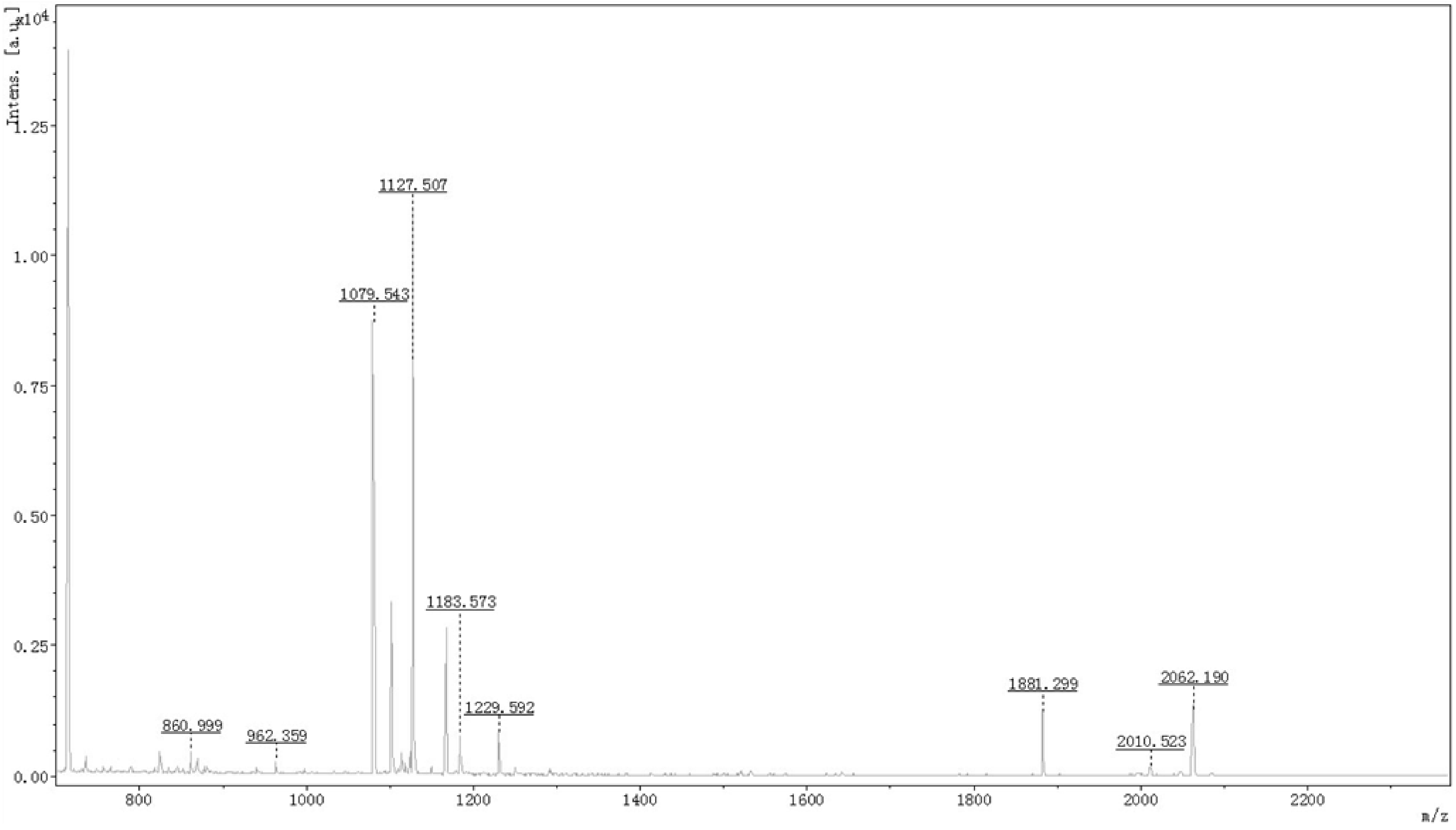


**Fig. S7** Mass spectrum of a trypsin-digested protein of putative outer membrane porin isolated from *K. pneumoniae* ATCC 13883. 713.5, 1,079.6, 1,101.6, 1,127.5, 1,166.6, 1,183.6, 1,229.6, 1,881.1 and 2,061.9-m/z peaks were chosed for the following MALDI-TOF/TOF MS analysis.

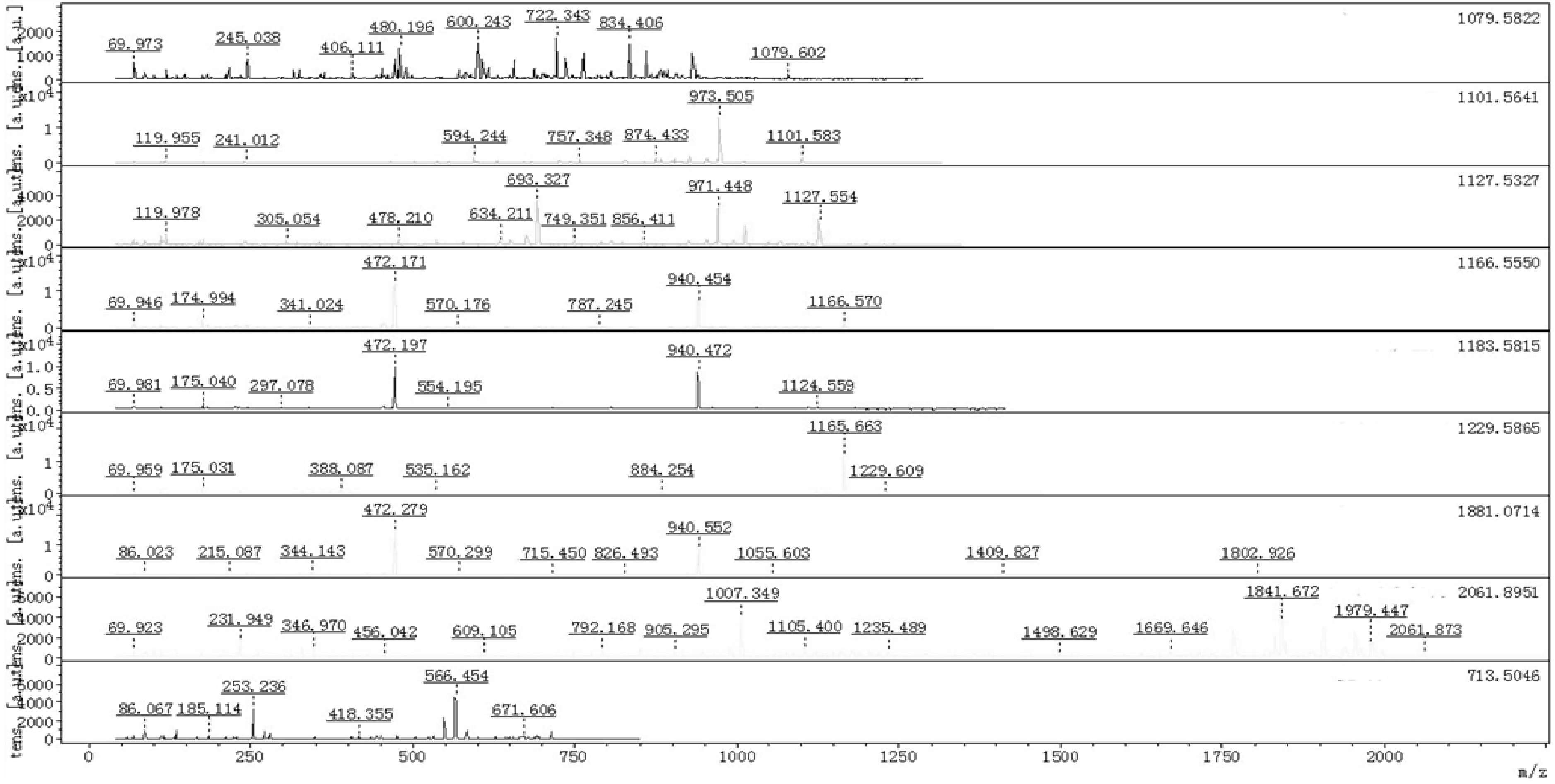


**Fig. S8** Mass spectra of nine tryptic peptides corresponding to putative outer membrane porin of *K. pneumoniae* ATCC 13883 by MALDI-TOF/TOF MS.
